# Supplementary material for: Luteinizing Hormone Receptor Is Expressed in Testicular Germ Cell Tumors: Possible Implications for Tumor Growth and Prognosis
Source: Cancers (Basel). 2020 May 26;12(6):1358. doi: 10.3390/cancers12061358 (PMC7352821; doi:10.3390/cancers12061358)
Supplement: Supplementary file 1 [file cancers-12-01358-s001.zip › cancers-788970-suppl-final/cancers-788970-suppl-final.docx]

Article

Luteinizing Hormone Receptor Is Expressed in Testicular Germ Cell Tumors: Possible Implications for Tumor Growth and Prognosis

Mette Lorenzen, John Erik Nielsen, Christine Hjorth Andreassen, Anders Juul, Birgitte Grønkær Toft, Ewa Rajpert-De Meyts, Gedske Daugaard, and Martin Blomberg Jensen

Supplementary Materials

**Table S1.** Antibodies and conditions used for IHC and WB.

| **Antibody** | **Species** | **Dilution IHC** | **Dilution WB** | **Retrieval**  **Buffer (IHC)** | **Antibody supplier and product name** | **Expected Molecular Weight** |
| --- | --- | --- | --- | --- | --- | --- |
| LHCGR (LHR029)  Extracelluar region | Mouse | 1:200 | 1:200 | TEG | NBCL  LHR029 | 79 kDa |
| LHCGR  Internal region | Goat | - | 1:200 | - | Santa Cruz  SC-26341 | 85 kDa |
| LHCGR (LHRsc)  Extracellular region | Rabbit | 1:400 | - | TEG | Santa Cruz  SC-25828 | - |
| LHCGR  C-terminal | Rabbit | 1:3000 | 1:200 | TEG | Aviva System Biology  OASG04237 | 80 kDa |
| B2M | Rabbit | - | 1:200 | - | Dako A0072 | 12 kDa |
| D2-40 | Mouse | 1:10.000 | - | TEG | Dako  M3619 | - |
| SOX2 | Goat | 1:1800 | - | TEG | R&D Systems  AF2018 | - |
| β-actin | Mouse | - | 1:200 | - | Santa Cruz  sc-8035 | 43 kDa |

Abbreviations*:* B2M, beta-2-microglobulin; IHC, immunohistochemistry; LHCGR, luteinizing hormone/choriogonadotropin receptor; SOX2, sex determining region Y-box 2; WB, western blot. For all antibodies, antigen retrieval was conducted by placing the sections in a pressure cooker in the indicated retrieval buffer for 60 min. TEG buffer: 10 mM.
