# Supplementary material for: Luteinizing Hormone Receptor Is Expressed in Testicular Germ Cell Tumors: Possible Implications for Tumor Growth and Prognosis
Source: Cancers (Basel). 2020 May 26;12(6):1358. doi: 10.3390/cancers12061358 (PMC7352821; doi:10.3390/cancers12061358)
Supplement: Supplementary file 1 [file cancers-12-01358-s001.zip › cancers-788970-suppl-final/Original WB figures .pdf]

Western blot - tissues

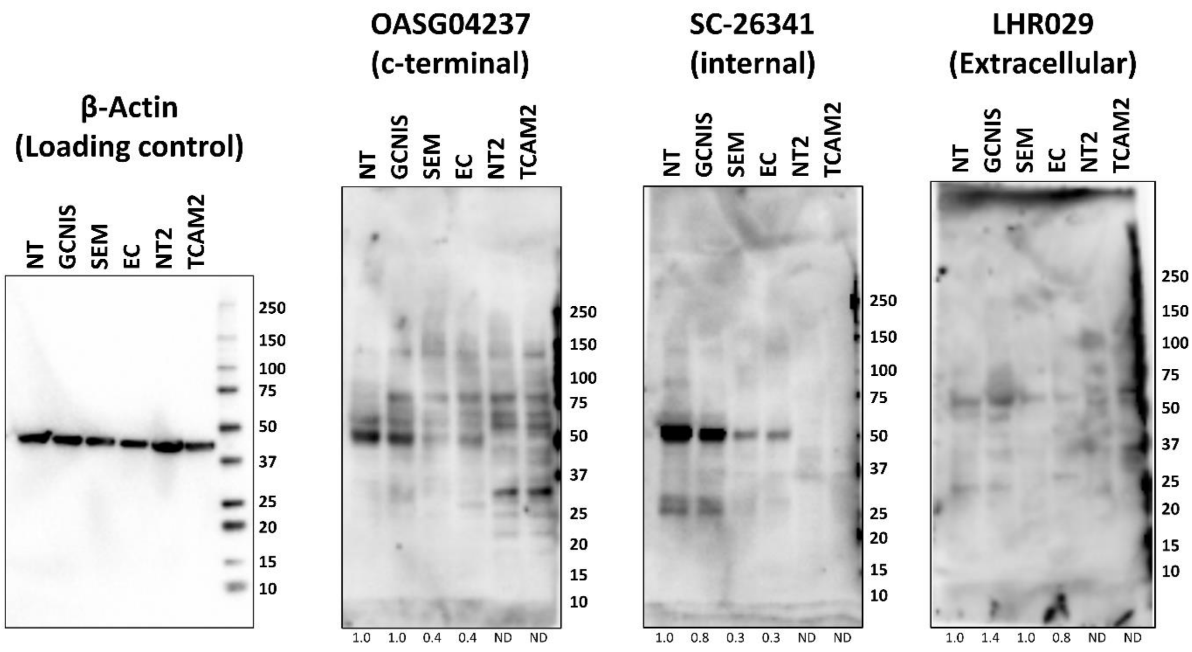

Western blot - Serum

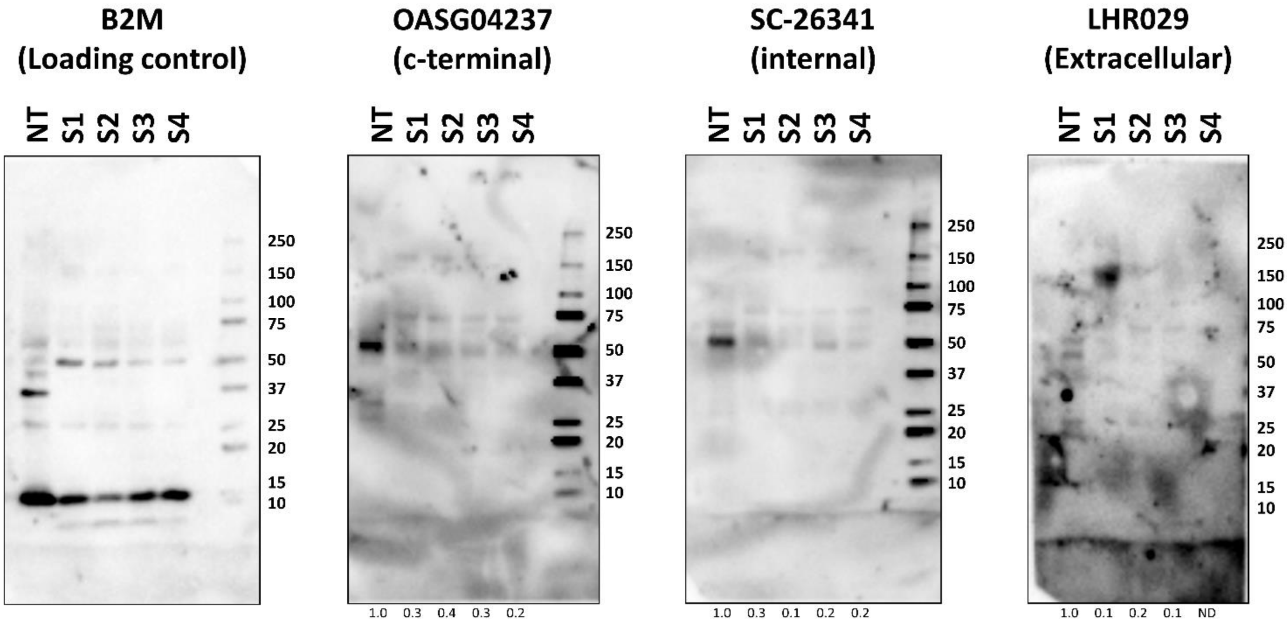

Quantification of the 50 kDA band is indicated below each Western blot figure and was performed relative to β-Actin or B2M and normalized to NT using ImageJ software.
